# Supplementary material for: Unveiling the Bioactive Potential of the Invasive Jellyfish Phyllorhiza punctata Through Integrative Transcriptomic and Proteomic Analyses
Source: Biomolecules. 2025 Aug 4;15(8):1121. doi: 10.3390/biom15081121 (PMC12383608; doi:10.3390/biom15081121)
Supplement: Supplementary file 1 [file biomolecules-15-01121-s001.zip › Suplementary Material/FileS3/PEPFOLD4/XP_065055704_15191/clusters.html]

 PEP-FOLD clusters


Download archive of all models  here   

|  |  |  |
| --- | --- | --- |
| # | file | sOPEP |
| # cluster 1 - Size: 2 |  |  |
|  | XP\_065055704\_15191-model1 | -6.43654 |
|  | XP\_065055704\_15191-model1.1 | -5.0242 |
| # cluster 2 - Size: 15 |  |  |
|  | XP\_065055704\_15191-model2 | -5.434 |
|  | XP\_065055704\_15191-model2.1 | -5.434 |
|  | XP\_065055704\_15191-model2.2 | -5.18484 |
|  | XP\_065055704\_15191-model2.3 | -5.11047 |
|  | XP\_065055704\_15191-model2.4 | -5.05596 |
|  | XP\_065055704\_15191-model2.5 | -4.89772 |
|  | XP\_065055704\_15191-model2.6 | -4.77439 |
|  | XP\_065055704\_15191-model2.7 | -4.53412 |
|  | XP\_065055704\_15191-model2.8 | -4.49234 |
|  | XP\_065055704\_15191-model2.9 | -4.43423 |
|  | XP\_065055704\_15191-model2.10 | -4.39677 |
|  | XP\_065055704\_15191-model2.11 | -4.28712 |
|  | XP\_065055704\_15191-model2.12 | -4.17842 |
|  | XP\_065055704\_15191-model2.13 | -4.16942 |
|  | XP\_065055704\_15191-model2.14 | -4.12381 |
| # cluster 3 - Size: 9 |  |  |
|  | XP\_065055704\_15191-model3 | -5.10149 |
|  | XP\_065055704\_15191-model3.1 | -5.06054 |
|  | XP\_065055704\_15191-model3.2 | -4.50827 |
|  | XP\_065055704\_15191-model3.3 | -4.39382 |
|  | XP\_065055704\_15191-model3.4 | -4.33671 |
|  | XP\_065055704\_15191-model3.5 | -4.28765 |
|  | XP\_065055704\_15191-model3.6 | -4.18814 |
|  | XP\_065055704\_15191-model3.7 | -4.14525 |
|  | XP\_065055704\_15191-model3.8 | -4.08582 |
| # cluster 4 - Size: 1 |  |  |
|  | XP\_065055704\_15191-model4 | -4.96235 |
| # cluster 5 - Size: 7 |  |  |
|  | XP\_065055704\_15191-model5 | -4.90854 |
|  | XP\_065055704\_15191-model5.1 | -4.84445 |
|  | XP\_065055704\_15191-model5.2 | -4.47647 |
|  | XP\_065055704\_15191-model5.3 | -4.447 |
|  | XP\_065055704\_15191-model5.4 | -4.43552 |
|  | XP\_065055704\_15191-model5.5 | -4.31553 |
|  | XP\_065055704\_15191-model5.6 | -4.05589 |
| # cluster 6 - Size: 22 |  |  |
|  | XP\_065055704\_15191-model6 | -4.47196 |
|  | XP\_065055704\_15191-model6.1 | -4.44509 |
|  | XP\_065055704\_15191-model6.2 | -4.41462 |
|  | XP\_065055704\_15191-model6.3 | -4.40003 |
|  | XP\_065055704\_15191-model6.4 | -4.3873 |
|  | XP\_065055704\_15191-model6.5 | -4.34064 |
|  | XP\_065055704\_15191-model6.6 | -4.30897 |
|  | XP\_065055704\_15191-model6.7 | -4.2793 |
|  | XP\_065055704\_15191-model6.8 | -4.25287 |
|  | XP\_065055704\_15191-model6.9 | -4.25056 |
|  | XP\_065055704\_15191-model6.10 | -4.23558 |
|  | XP\_065055704\_15191-model6.11 | -4.22728 |
|  | XP\_065055704\_15191-model6.12 | -4.22204 |
|  | XP\_065055704\_15191-model6.13 | -4.2007 |
|  | XP\_065055704\_15191-model6.14 | -4.2007 |
|  | XP\_065055704\_15191-model6.15 | -4.19949 |
|  | XP\_065055704\_15191-model6.16 | -4.18254 |
|  | XP\_065055704\_15191-model6.17 | -4.16458 |
|  | XP\_065055704\_15191-model6.18 | -4.14388 |
|  | XP\_065055704\_15191-model6.19 | -4.12889 |
|  | XP\_065055704\_15191-model6.20 | -4.12204 |
|  | XP\_065055704\_15191-model6.21 | -4.06022 |
| # cluster 7 - Size: 4 |  |  |
|  | XP\_065055704\_15191-model7 | -4.47139 |
|  | XP\_065055704\_15191-model7.1 | -4.3071 |
|  | XP\_065055704\_15191-model7.2 | -4.08086 |
|  | XP\_065055704\_15191-model7.3 | -4.07395 |
| # cluster 8 - Size: 14 |  |  |
|  | XP\_065055704\_15191-model8 | -4.39274 |
|  | XP\_065055704\_15191-model8.1 | -4.33472 |
|  | XP\_065055704\_15191-model8.2 | -4.29265 |
|  | XP\_065055704\_15191-model8.3 | -4.28973 |
|  | XP\_065055704\_15191-model8.4 | -4.25203 |
|  | XP\_065055704\_15191-model8.5 | -4.24524 |
|  | XP\_065055704\_15191-model8.6 | -4.21961 |
|  | XP\_065055704\_15191-model8.7 | -4.20939 |
|  | XP\_065055704\_15191-model8.8 | -4.19932 |
|  | XP\_065055704\_15191-model8.9 | -4.1737 |
|  | XP\_065055704\_15191-model8.10 | -4.16983 |
|  | XP\_065055704\_15191-model8.11 | -4.15664 |
|  | XP\_065055704\_15191-model8.12 | -4.03194 |
|  | XP\_065055704\_15191-model8.13 | -4.01457 |
| # cluster 9 - Size: 5 |  |  |
|  | XP\_065055704\_15191-model9 | -4.39091 |
|  | XP\_065055704\_15191-model9.1 | -4.32512 |
|  | XP\_065055704\_15191-model9.2 | -4.3143 |
|  | XP\_065055704\_15191-model9.3 | -4.24903 |
|  | XP\_065055704\_15191-model9.4 | -4.22069 |
| # cluster 10 - Size: 6 |  |  |
|  | XP\_065055704\_15191-model10 | -4.29641 |
|  | XP\_065055704\_15191-model10.1 | -4.19717 |
|  | XP\_065055704\_15191-model10.2 | -4.16739 |
|  | XP\_065055704\_15191-model10.3 | -4.16498 |
|  | XP\_065055704\_15191-model10.4 | -4.14821 |
|  | XP\_065055704\_15191-model10.5 | -4.14821 |
| # cluster 11 - Size: 3 |  |  |
|  | XP\_065055704\_15191-model11 | -4.26398 |
|  | XP\_065055704\_15191-model11.1 | -4.22795 |
|  | XP\_065055704\_15191-model11.2 | -4.16547 |
| # cluster 12 - Size: 2 |  |  |
|  | XP\_065055704\_15191-model12 | -4.21529 |
|  | XP\_065055704\_15191-model12.1 | -4.21366 |
| # cluster 13 - Size: 10 |  |  |
|  | XP\_065055704\_15191-model13 | -4.15565 |
|  | XP\_065055704\_15191-model13.1 | -4.15084 |
|  | XP\_065055704\_15191-model13.2 | -4.13874 |
|  | XP\_065055704\_15191-model13.3 | -4.13606 |
|  | XP\_065055704\_15191-model13.4 | -4.13171 |
|  | XP\_065055704\_15191-model13.5 | -4.12927 |
|  | XP\_065055704\_15191-model13.6 | -4.11174 |
|  | XP\_065055704\_15191-model13.7 | -4.06019 |
|  | XP\_065055704\_15191-model13.8 | -4.04304 |
|  | XP\_065055704\_15191-model13.9 | -4.01764 |
|  |  |  |
